# At clinically relevant concentrations the anaesthetic/amnesic thiopental but not the anticonvulsant phenobarbital interferes with hippocampal sharp wave-ripple complexes

**DOI:** 10.1186/1471-2202-8-60

**Published:** 2007-07-31

**Authors:** Costas Papatheodoropoulos, Evangelos Sotiriou, Dimitrios Kotzadimitriou, Panagiota Drimala

**Affiliations:** 1 Department of Physiology, Medical School, University of Patras, Rion, Greece; 2 Division of Basic Neurosciences, Foundation for Biomedical Research of the Academy of Athens (IIBEAA), Athens, Greece; 3 Central and North West London Mental Health NHS Trust, Substance Misuse Service, 5-7 Wolverton Gardens, London, W6 7DY, UK

## Abstract

**Background:**

Many sedative agents, including anesthetics, produce explicit memory impairment by largely unknown mechanisms. Sharp-wave ripple (SPW-R) complexes are network activity thought to represent the neuronal substrate for information transfer from the hippocampal to neocortical circuits, contributing to the explicit memory consolidation. In this study we examined and compared the actions of two barbiturates with distinct amnesic actions, the general anesthetic thiopental and the anticonvulsant phenobarbital, on in vitro SPW-R activity.

**Results:**

Using an in vitro model of SPW-R activity we found that thiopental (50–200 μM) significantly and concentration-dependently reduced the incidence of SPW-R events (it increased the inter-event period by 70–430 %). At the concentration of 25 μM, which clinically produces mild sedation and explicit memory impairment, thiopental significantly reduced the quantity of ripple oscillation (it reduced the number of ripples and the duration of ripple episodes by 20 ± 5%, n = 12, *P *< 0.01), and suppressed the rhythmicity of SPWs by 43 ± 15% (n = 6, *P *< 0.05). The drug disrupted the synchrony of SPWs within the CA1 region at 50 μM (by 19 ± 12%; n = 5, *P *< 0.05). Similar effects of thiopental were observed at higher concentrations. Thiopental did not affect the frequency of ripple oscillation at any of the concentrations tested (10–200 μM). Furthermore, the drug significantly prolonged single SPWs at concentrations ≥50 μM (it increased the half-width and the duration of SPWs by 35–90 %). Thiopental did not affect evoked excitatory synaptic potentials and its results on SPW-R complexes were also observed under blockade of NMDA receptors. Phenobarbital significantly accelerated SPWs at 50 and 100 μM whereas it reduced their rate at 200 and 400 μM. Furthermore, it significantly prolonged SPWs, reduced their synchrony and reduced the quantity of ripples only at the clinically very high concentration of 400 μM, reported to affect memory.

**Conclusion:**

We hypothesize that thiopental, by interfering with SPW-R activity, through enhancement of the GABA_A _receptor-mediated transmission, affects memory processes which involve hippocampal circuit activation. The quantity but not the frequency of ripple oscillation was affected by the drug.

## Background

Sedative drugs, including barbiturates, induce episodic memory impairment in humans [1-4] through poorly understood mechanisms. The hippocampus appears to be a key structure in episodic memory processing [5-7]. Timely structured activity of cell assemblies is thought to underlie encoding and transient storage of explicit memory traces in the hippocampal circuit [8]. These memory traces are reactivated during subsequent behavioral stages [9-11] namely during bursts of activity called sharp wave-ripple complexes (SPW-R) consisting of a relatively slow wave (sharp wave; [12-14] crowned by high frequency oscillation (ripple, 100–200 Hz; [13,15-17]. SPW-R activity occurs mainly during slow wave sleep and awake immobility [13,18], and it is also observed in high temporal proximity to the experienced event [19]. Studies showing temporal correlation between SPW-R and neocortical activity [17,20,21] suggest that SPW-R constitute the neuronal substrate for information transfer in the hippocampo-neocortical circuits [22], contributing to the long-term storage of short-term memories and thus to memory consolidation in neocortical circuits [17,23,24]. The study of the effects of amnesia-inducing agents on hippocampal SPW-R activity becomes increasingly interesting in light of the above research evidence.

In vitro experimentation continuously provides new data demonstrating the great impact of isolated preparations as models in elucidating the mechanisms underlying brain rhythms and the action of centrally acting drugs on these rhythms [25,26]. In particular, slice preparation offers unique advantages of experimental control for studying the network mechanisms underlying distinct and isolated elements of cooperative population activities. In vitro models of SPW-R activity have recently been developed using hippocampal preparations [27-31]. In vitro SPW-R activity presents certain experimental advantages including its spontaneous generation in hippocampal slices under standard conditions without being induced by pharmacological agents or other experimental manipulation. Thus, it is especially suitable for pharmacological examinations since complex drug interactions which could interfere with the mechanisms underlying the effects of the drug under investigation are avoided.

Using the in vitro SPW-R we studied the effects of two barbiturates, thiopental and phenobarbital, which have different clinical usage and display distinct effects on memory processes. Thiopental, one of the most commonly used barbiturates, is an intravenously used general anesthetic, producing anterograde amnesia [32-34]. It preferentially affects explicit memory [1,35]. General anesthetics apparently affect the transition from short-term to long-term storage of explicit memories [36]. On the other hand, the anticonvulsant phenobarbital shows less consistent effects on memory processes. Some studies revealed no detectable effects on memory [37,38] while others describe memory impairment [39,40] especially when doses at the high end of the therapeutic range were used [41,42].

We show that at clinically relevant concentrations the two drugs differently affect SPW-R complexes, with thiopental strongly affecting activity and phenobarbital displaying no significant effects.

## Results

### Characteristics of SPW-R activity

**Figure 1:**
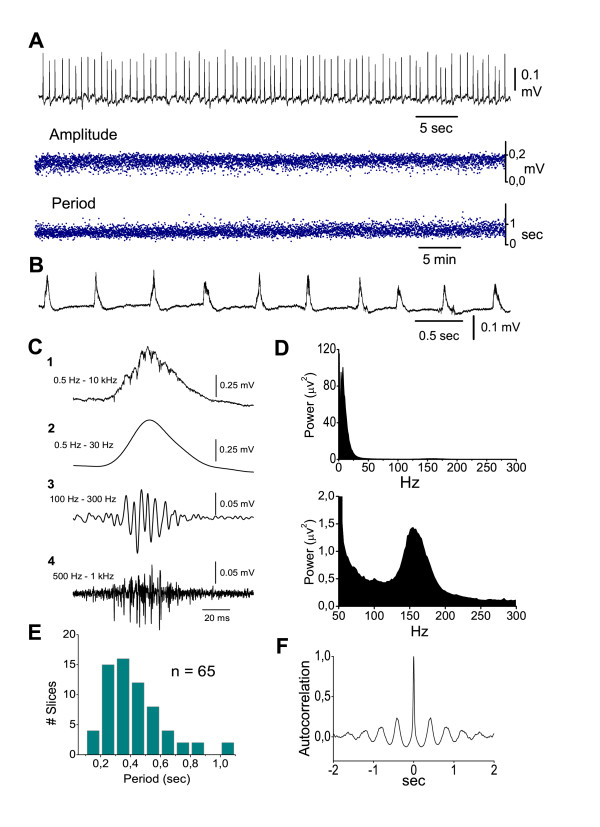
**In vitro SPW-R activity recorded in the st. pyramidale of CA1 region of ventral hippocampal slices perfused with standard medium**. **A**. Continuous long-lasting field recording of SPWs (trace on the top, low-pass filtered at 35 Hz) and the corresponding time histograms of amplitude (middle) and period (bottom). Data were obtained from a slice four hours after its placement on the recording chamber. Note the persistency and stability over time of both characteristics of SPWs. **B**. Record of spontaneous activity obtained from a different slice, presented at a faster sweep speed. **C**. A wide band record of a single SPW-R event (C1) and the filtered sweeps are shown, illustrating the distinct components of the event. Low-pass (0.5–30 Hz) and band-pass (100–300 Hz) filtering reveals the slow wave (C2) and the associated ripple oscillation (C3) respectively. Band-pass filtering at very high frequencies (0.5–1 kHz, C4) discloses the burst-like multiunit activity occurring mostly during the rising and peaking phases of the slow positive potential. **D**. FFT from 1 min raw record of spontaneous activity showing the difference in power between SPWs (upper diagram) and ripples (lower diagram, same power spectrum shown at a greater magnification). SPW dominant peak is at 5–10 Hz. Ripple activity is at ~150 Hz. **E**. Histogram of period (inter-event interval) of SPWs calculated from a population of slices, showing that most of the slices displayed a period between 0.2–0.6 sec, which correspond to a frequency range of 1.5–5.0 Hz. **F**. Auto-correlogram from a 5 min low-pass record from a distinct experiment, showing the typical degree of rhythmicity of SPWs.

**Figure 2:**
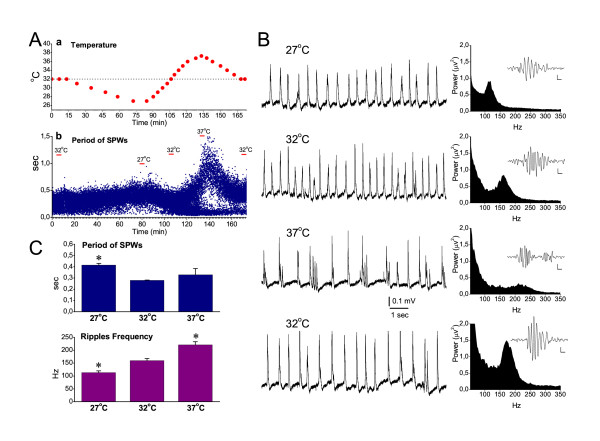
**Effects of temperature on SPWs and ripple oscillation**. **A**. Representative experiment showing the time course of the temperature changes (a) and the induced fluctuations in the period of SPWs recorded from the CA1 pyramidal layer (b). The dotted line in "a" marks the temperature of 32°C. In "b", the three representative temperatures (32°C, 27°C and 37°C) are indicated for purpose of comparison. During the increase of the temperature from 34–35°C to higher values, the histogram of the period bifurcated. This deviation of the values was due to the fact that SPWs were frequently generated in a burst-like fashion (shown in B), where consecutive waves appeared at short intervals, whereas at the same time the period between bursts of events increased. **B**. Continuous recording of SPW-R complexes (left column) and power spectra at the high frequency range (right column) obtained at different temperatures. Single ripple episodes (band-pass filtered traces) are shown as inserts in spectral plots; calibration bars: 10 μV, 10 ms. At 37°C SPWs appeared frequently as bursts of several events. Ripple frequency changed monotonically with temperature. Note that at 37°C ripple frequency exceeded 200 Hz. Data in "A" an "B" were collected from two different slices. **C**. Collective date of the period of SPWs and of the frequency of ripple oscillation at three different temperatures. Asterisks indicate significant differences with reference to the group of 32°C (paired t-test, at *P *< 0.05, n = 4).

**Figure 3:**
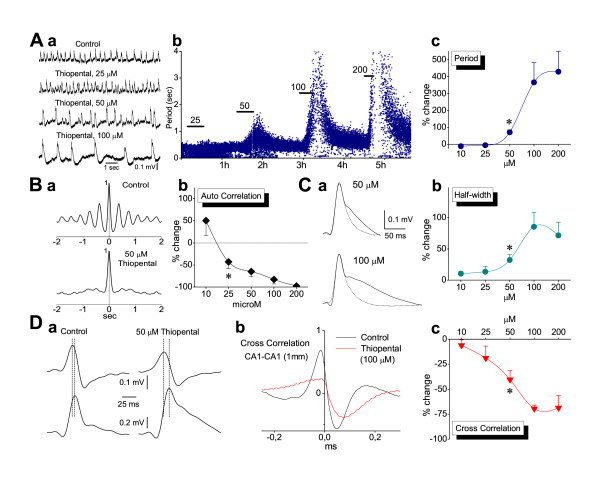
**Effects of thiopental on Sharp Waves**. **A**. Example of field recordings (a) and plot of instantaneous period (b) illustrating that thiopental induced a concentration-dependent and reversible increase in the inter-event period. Horizontal bars in "b" denote the time of application of consecutively larger drug concentrations. Relatively large data values of period during 100 and 200 μM of the drug are curtailed for reasons of clarity of lower values. Note the great value dispersion of instant period during drug application at ≥50 μM. Data in "a" and "b" were obtained from two different slices. The collective data plot (percent changes) of period is shown in (c). The asterisk here, as in the subsequent collective plots in the figure, denotes the lowest concentration with statistically significant drug action (paired t-test, *P *< 0.001 at 50 μM and *P *< 0.05 at 100–200 μM). The number of slices studied at the five concentrations, of 10, 25, 50, 100 and 200 μM, were respectively 12, 18, 22, 12 and 8. Error bars not shown were smaller than the size of the symbol. **B**. Example of auto-correlation before and during 50 μM thiopental (a) and collective auto-correlation plot (b) showing the disruption of rhythmicity at concentrations ≥25 μM (paired t-test, *P *< 0.05 at 25 μM and *P *< 0.01 at ≥50 μM). Numbers of slices studied at the five concentrations were 5, 6, 12, 8 and 4. **C**. Example of thiopental-induced prolongation of SPWs (a) at two different concentrations. Averages of low-pass filtered sweeps taken from a two minute epoch are shown. Dotted traces are control sweeps. The corresponding collective plot (b) shows that significant prolongation of single SPWs occurred at drug concentrations ≥50 μM (paired t-test, *P *< 0.01). **D**. Effect of thiopental on SPWs' synchronization along the CA1 region. Single SPWs simultaneously recorded from two locations along the CA1 st. pyramidale, measuring 1 mm, are shown in "a", before (traces on the left) and during application of 50 μM thiopental (traces on the right). Sweeps were low-pass filtered in order to make feasible the time-discrimination of their peaks. Note the increased phase-lag (marked by dotted lines) during drug application (9.25 ms) compared with control condition (3.8 ms). The cross-correlation plot from another slice (b) illustrates the large decrease of function value during drug application at 100 μM. The collective plot (c) shows that significant suppression of synchrony started at 25 μM and reached a plateau at 100 μM (*P *< 0.05 at 50 μM and *P *< 0.01 at ≥50 μM). Numbers of slices studied at the five concentrations were 3, 5, 5, 4 and 4.

**Figure 4:**
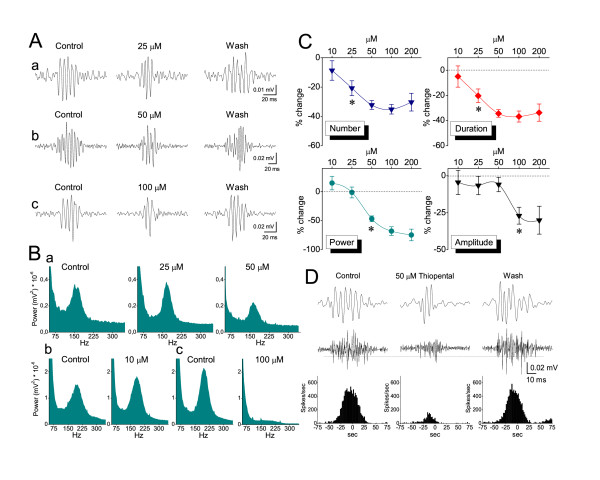
**Effects of thiopental on Ripple oscillation**. **A**. Examples of ripple oscillation (filtered traces) from three slices (a-c) illustrating the concentration-dependent reversible reduction in number of ripples and duration of ripple episode by thiopental. Note that even at 100 μM of the drug, which strongly suppressed the amount of ripples, the oscillation frequency remained unchanged. **B**. Power spectra from three experiments (a-c) showing the reduction of ripple power at 50 and 100 μM but not at lower concentrations. **C**. Plots of collective data of drug effects (percent changes) on the various measures of ripple oscillation. Asterisks mark the lowest concentration with statistically significant drug action (see text for details of statistical analysis). Note that ripple number and ripple episode duration were more sensitive than ripple power and ripple amplitude. Measures of number, duration and amplitude for each of the five drug concentrations (10, 25, 50, 100 and 200 μM) were respectively collected from 10, 12, 17, 10 and 8 slices. The corresponding numbers of slices for ripple power measures were respectively 12, 14, 22, 15, and 9. **D**. Recordings of ripples (upper traces) and multiunits (middle traces), revealed after filtering original records, showing the thiopental-induced reduction in the rate of multiunits during ripple episodes. Dotted line indicates the threshold level set in this experiment for spike detection. Histograms of multiunit rate (bottom panel) triggered by peaks of SPWs (not shown) used to measure the mean rate under the different experimental conditions.

Spontaneous sharp wave-ripple (SPW-R) complexes (Fig. 1A,B) were consistently recorded in seventy-eight ventral hippocampal slices taken from forty-eight animals. SPWs in st. pyramidale consisted of positive potentials with a mean amplitude, half-width and duration of 0.22 ± 0.02 mV (n = 74), 24.8 ± 6.5 ms (n = 60) and 79.8 ± 5.4 ms (n = 60) respectively, and they occurred every 0.410 ± 0.02 sec (n = 74) (Fig. 1B–C,E). SPWs although not literally oscillatory, presented a certain degree of rhythmicity evident using auto-correlation analysis [31], (Fig. 1F). Ripples appeared as a high-frequency oscillation associated to the SPW slow potential and they were detected after band-pass filtering raw signal at 100–300 Hz (Fig. 1C). Ripple oscillation was also revealed as a clearly distinguishable peak in fast Fourier transforms (Fig. 1D). The mean values of the ripple number inside a ripple episode, the duration of ripple episode and the frequency of ripple oscillation were respectively 7.0 ± 0.3 ripples (n = 49), 38 ± 2 ms (n = 49) and 161.0 ± 2.5 Hz (n = 69). Multiunit activity, revealed after band-pass filtering, was associated with SPW-R events (Fig. 1C-4). Similarly to previous observations [43] the mean frequency of multiunit activity accompanying SPW-Rs ranged among slices from 75 to 420 Hz (mean value 232 ± 36 Hz, n = 9 slices). Most of the experiments were carried out in CA1; however, in some experiments recordings were made in CA3. Statistical comparisons (independent t-test) showed that the different measures did not significantly differ between the two fields. For instance, in CA1 the mean values of amplitude, half-width and duration of SPWs were respectively 0.21 ± 0.02 mV (n = 63), 25.0 ± 1.0 ms (n = 46) and 79.0 ± 6.3 ms (n = 46); the corresponding values in CA3 (n = 11) were respectively 0.27 ± 0.05 mV, 24 ± 1.2 ms and 83.0 ± 9.9 ms. The measures of ripples were similar between CA1 and CA3 as well, although the ripple episodes were slightly longer in CA1 than in CA3, but not significantly so. The mean values of the ripples' number, the duration of the ripple episode and the ripple frequency in CA1 (n = 31) vs CA3 (n = 8) were respectively 7.4 ± 0.4 vs 6.7 ± 0.6 ripples, 41.2 ± 2.7 vs 36.3 ms and 161.0 ± 3.3 vs 164.0 ± 6.5 Hz.

The pharmacological experiments in the present study were carried out at 32°C, which is 4–7°C lower than the body temperature in the intact animal [44]. In order to examine the effects of temperature on SPWs and ripple oscillation, the temperature in the recording chamber was changed systematically while continuously recording SPW-R activity from the CA1 pyramidal layer. The temperature changes significantly affected the rate of SPWs and the ripple frequency, but not the magnitude (i.e. amplitude or power) of the events. Furthermore, SPWs and ripples were affected differently. In particular, lowering the temperature from 32 to 27°C significantly slowed down SPWs by 49 ± 6 % (paired t-test, n = 4, *P *< 0.01) (Fig. 2). The mean rate of SPWs was not significantly changed when the temperature was increased from 32°C to 37°C, as has been also previously observed [45]. However, at 37°C SPWs were often generated as bursts of two to four waves (Fig. 2B). The ripple frequency changed linearly with temperature, increasing monotonically when the temperature was increased from 27 to 37°C. In particular, the ripple frequency increased by 29.4 ± 0.7 % from 27 to 32°C and by 38 ± 4 % from 32 to 37°C (Fig. 2B,C). The mean value of the ripple frequency observed at 32°C (160 ± 8 Hz) was at the range of the ripple frequency observed in vivo (i.e. 120–200 Hz) [15,46-48]. However, at 27°C and 37°C the values of ripple frequency (113 ± 6.6 Hz and 221 ± 12.5 Hz respectively) were at the limits of the physiological range of the ripple frequency. These experiments indicated that, although certain properties of SPW-Rs in vitro are affected by temperature, in vitro SPW-R activity closely resembles its in vivo counterpart when slices are maintained at the temperature of 32°C.

### Thiopental effects on in vitro SPW-R complexes

Thiopental was applied in the perfusion medium at the concentrations of 10, 25, 50, 100 and 200 μM. Clinical concentrations of thiopental range from 10 to about 100 μM [1,32,35,49-51]. Somewhat larger maximal concentrations have been reported in studies using experimental animals [52-55]. Recordings were typically performed in CA1, but in some experiments (no more than three at each concentration) recordings were made in CA3. Since no difference was found between CA1 and CA3 the measures obtained from the two fields were pooled together. Thiopental produced concentration-dependent and reversible changes in most of the characteristics of SPWs and ripples. As illustrated in Fig. 3, thiopental at concentrations ≥50 μM significantly reduced the rate of SPWs (i.e. it increased the period between single SPWs) and prolonged single SPW (Fig. 2A &2C), without significantly affecting the amplitude of potentials, the mean percent change of which at the five concentrations (10, 25, 50 100 and 200 μM) was respectively 1.6 ± 5.8% (n = 12), 0.4 ± 4.3% (n = 18), 16.4 ± 8.5% (n = 21), 13.3 ± 12.2% (n = 12) and 10.3 ± 18.7% (n = 9). Furthermore, the drug significantly reduced rhythmicity of SPWs (starting at 25 μM; Fig. 3B) and the synchronization of SPWs across the CA1 region, as observed by the reduction in cross-correlation (Fig. 3D). At the concentrations of 100 and 200 μM the drug abolished spontaneous activity in 4/15 and 5/12 slices respectively.

Thiopental at a concentration-dependent manner shortened ripple episodes by reducing the number of ripples in each episode, without changing the ripple frequency (Fig. 4). Taking into account that thiopental prolonged single SPW it resulted that less of the SPW waveform was accompanied by a ripple episode following drug application. Thiopental significantly reduced the amplitude and the power of ripple oscillation. The ripple number and the duration of ripple episode were proven the most sensitive measures, with the drug significantly affecting these at the concentration onset of 25 μM (paired t-test, *P *< 0.01) and reaching maximal effect values at the concentration of 50 μM (*P *< 0.001; Fig. 4C). The power of ripple oscillation was significantly reduced at concentrations ≥50 μM (*P *≤ 0.01), whereas the amplitude was significantly affected at concentrations of 100 and 200 μM (*P *< 0.05; Fig. 4C). Even at high concentrations thiopental only induced a small, statistically non significant reduction in the frequency of the oscillation (maximal reduction by 17 ± 2.6 % at 100 μM). It is interesting that at the lowest concentration used (10 μM), the drug produced a consistent, yet statistically insignificant increase in the power of ripple oscillation (by 14.87 ± 11.6 %; Fig. 4B-b,C), and a small acceleration of the rate of SPWs (reduction in period by 11.1 ± 4.5 %; Fig. 3C), reminiscent of the EEG-activation at very light levels of general anesthesia [54].

It has been shown that thiopental reduces neuronal excitability [56]. In order to examine the action of the thiopental on the neuronal excitability, we measured SPW-R-associated multiunit activity before and during application of the drug [57]. The calculation of rate was made using the same time window at control and drug conditions. The duration of the window equated to the time around the maximum rate value, which included the 75% of the multiunit rate histogram. Thus, differences in the rate could be attributed to actual changes in the rate rather than to the total number of units fired during an SPW-R episode, taken into account that the drug induced shortening of SPW-R episodes. Thiopental produced significant and reversible reduction in the mean rate of multiunit activity by 43.8 ± 6.5 % (n = 9; *P *< 0.05) (Fig. 4D).

**Figure 5:**
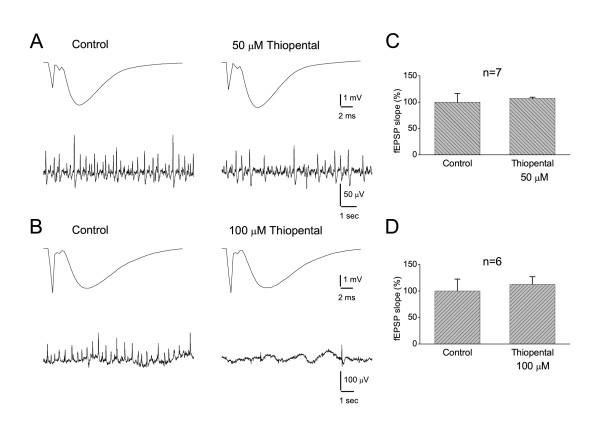
**Thiopental did not affect fEPSPs**. **A-B**. Evoked fEPSPs from CA1 st. radiatum (upper traces, averages of ten sweeps) and continuous records of SPW-Rs from CA1 st. pyramidale (lower traces) obtained in normal medium (left panels) and in medium containing 50 or 100 μM of thiopental (right panels). fEPSPs and spontaneous activity were simultaneously recorded from two neighboring slices in "A" and from the same slice in "B". Note that the drug did not affect fEPSPs despite its suppressive effect on spontaneous activity. **C-D**. Grouped data of fEPSP slope for the two concentrations of thiopental. The drug did not significantly change the slope of fEPSP at any concentration.

Previous studies have shown that thiopental reduces excitatory synaptic events but only at concentrations greater than 50 μM [58-60]. We examined the effect of thiopental (50 and 100 μM) on the evoked field excitatory postsynaptic potential (fEPSP) recorded from the CA1 st. radiatum. Experiments were performed using the half-maximal fEPSP found on the base of input/output curve. In line with previous observations [58,60] we found that thiopental did not affect fEPSPs at 50 μM (Fig. 5). However, in contrast to previous studies [58,59,61] we did not observe any significant effect of thiopental at 100 μM.

**Figure 6:**
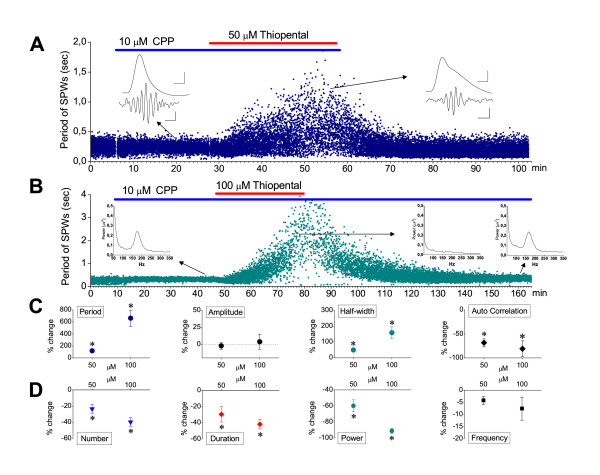
**Thiopental action on SPW-Rs does not involve NMDA-mediated synaptic transmission**. **A**. Representative histogram of instantaneous period of SPW-R activity recorded from the CA1 st. pyramidale. Thiopental (50 μM, red bar), applied in the presence of NMDA receptor antagonist CPP (10 μM, blue bar) produced reversible reduction in the rate of SPWs, a similar effect to that observed when the anesthetic was applied alone (compare with Fig. 3A). Inserts show examples of SPWs (traces on the top, low-pass filtered) and ripples (traces on the bottom, band-pass filtered) selected from times before and during thiopental application (arrows indicating the panel on the left and right respectively). Note that under thiopental SPWs are prolonged and ripple episode was shortened, containing decreased number of ripple waves. Calibration bars in the inserts: 50 μV, 25 ms for SPW; 25 μV, 50 ms for ripples. **B**. Histogram of instantaneous period of SPW-R activity recorded from the CA1 st. pyramidale of a slice which received 100 μM of thiopental (red bar) in the presence of CPP (blue bar). Inserts show the power spectra, at the ripple range, generated from epochs of 2 min taken from the different conditions (i.e. in CPP, in thiopental and after washing-out thiopental) as indicated by arrows. **C-D**. Collective data of the effects of thiopental on SPWs' (C) and Ripples' (D) measures in the presence of CPP. Measures of SPWs and Ripples were obtained from 9 and 7 slices respectively, at the concentration of 50 μM and from 8 and 5 slices respectively at the concentration of 100 μM. Asterisks indicate statistically significant drug effects (paired t-test, at *P *< 0.05). Error bars not shown were smaller than the size of the symbol.

Generation of SPW-Rs does not require activation of NMDA receptors [31,45,62-64]. However, conditions facilitating NMDA receptor activation, such as low magnesium medium, or direct exogenous activation of NMDA receptors, may affect SPW-R activity [64]. On the other hand, thiopental (50 μM) elicits burst suppression activity in the neocortex by a mechanism involving activation of NMDA receptors [58,65]. From a phenomenological point of view, there was a similarity between spontaneous activity observed in the present study under high drug concentrations and burst suppression activity (i.e. both are low-frequency high-voltage activity separated by periods of silence). Using recordings of SPW-R activity from the CA1 field, we examined whether NMDA receptors participate in the effects produced by thiopental at the concentrations of 50 or 100 μM. As shown in figure 6, in the presence of NMDA receptor antagonist CPP (10 μM), thiopental produced similar effects to those observed when the drug was administered alone, in standard medium. In particular, thiopental, at both concentrations (i.e. 50 and 100 μM) significantly increased the period of SPWs and prolonged single SPWs without affecting the amplitude of the waves and significantly reduced their rhythmicity (*P *< 0.05; n = 9 and n = 8 at 50 and 100 μM respectively) (Fig. 6A–C). Furthermore, the drug significantly reduced the quantity of ripples (i.e. their number, duration and power, *P *< 0.05; n = 7 and n = 5 at 50 and 100 μM respectively) without affecting the ripple frequency (Fig. 6D; examples shown in inserts of Fig. 6A &6B). At 100 μM, thiopental abolished SPW-R activity in two out of ten slices.

### Phenobarbital effects on in vitro SPW-R complexes

**Figure 7:**
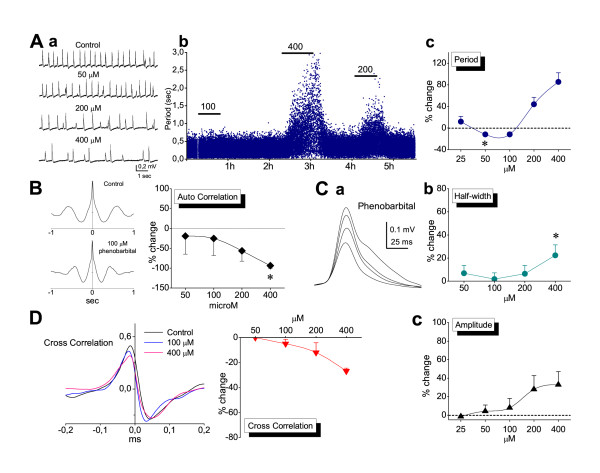
**Effects of phenobarbital on SPWs**. **A**. SPW-R complexes recorded from a slice bathed in normal medium and under consequently increasing concentrations of phenobarbital (a) showing that the rate of spontaneous activity was increased at 50 and 100 μM and reduced at 200 and 400 μM (asterisk denotes the lowest drug concentration with statistical significance effect, paired t-test, *P *< 0.001 paired t t-test, *P *< 0.05). In the plot of instantaneous period (b) note that even at the highest concentration (400 μM) phenobarbital did not abolish spontaneous activity. Measures for constructing the plot of collective data (c) were obtained from 3, 6, 10, 12 and 18 slices respectively for the five drug concentrations. **B**. Example of auto-correlation analysis for SPWs recorded in one slice before and during application of 100 μM of phenobarbital (diagram on the left). The collective data plot (diagram on the right) illustrates that only at 400 μM did the drug significantly reduce rhythmicity of SPWs (asterisk, *P *< 0.05). Data were obtained from 6, 10, 10 and 12 slices respectively for the four drug concentrations. **C**. Averages of filtered SPWs recorded from an epoch lasting two minutes (a) obtained from a slice bathed in normal medium (trace with the smallest amplitude) and increasing concentrations of phenobarbital (50, 200 and 400 μM, traces with consecutively increasing amplitude). Note the concomitant increase in the falling phase and the amplitude of the potentials. The drug significantly increased half-width of SPWs at the highest concentration of 400 μM (b). However, the drug did not significantly change the amplitude of SPWs at any concentration (c). Data in collective plots were obtained from 6, 10, 12 and 18 slices respectively for the four drug concentrations in half-width and from 3, 6, 10, 12 and 18 slices for the five concentrations in amplitude. **D**. Phenobarbital significantly decreased synchronization only at 400 μM, as illustrated in the example (on the left) and collective diagram (on the right, paired t-test, *P *< 0.05). The concentration of 25 μM is missing from the plots of auto-correlation, half-width and cross-correlation because measures of these variables were obtained from only one experiment.

**Figure 8:**
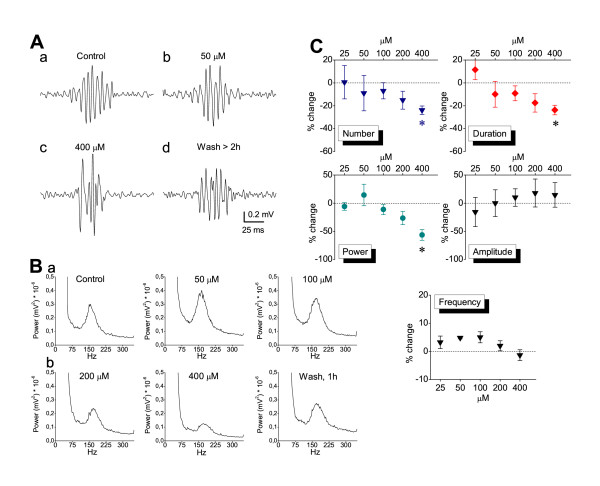
**Effects of phenobarbital on Ripple oscillation**. **A**. Examples of ripple oscillation obtained from one recording session showing that phenobarbital reversibly shortened ripple episode only at 400 μM (asterisks, paired t-tests, *P *< 0.05). **B**. Power spectra obtained from different times of a single experiment illustrating the reduction in ripple power only at high drug concentrations (200 and 400 μM). **C**. Diagrams of collective data for the different measures of the ripple oscillation. Note that the reducing effect of phenobarbital on number, duration and ripple power was statistically significant (asterisks, *P *< 0.05) only at the highest concentration of 400 μM. Measures of the above variables (except power) at the five drug concentrations were obtained from 2, 3, 7, 5 and 9 slices. Values of power were obtained from 4, 7, 12, 9 and 12 slices.

Phenobarbital was applied at the concentrations of 25, 50, 100, 200 and 400 μM and its effects on SPW-R activity were studied with recordings made from the CA1 field. Clinically relevant serum concentrations of phenobarbital range from 40 to 160 μM [66-68]. Free serum concentrations exceeding 100 μM produce severe sedation [69]. Given that cerebrospinal fluid concentration of phenobarbital is about half of the free serum levels [70], the maximum values of therapeutic (anticonvulsant) cerebrospinal fluid concentrations does not usually exceed 100 μM. As shown in the figures 7 and 8, phenobarbital at the concentration of 25 μM had no significant effect on SPW-R activity. At the concentrations of 50 and 100 μM it significantly accelerated the rate of SPWs without affecting any of the other characteristics of SPWs or the ripple oscillation. At concentrations greater than 100 μM the drug significantly slowed down the rate of SPWs (Fig. 7A). Significant effects on other measures were observed only at the highest concentration used (400 μM) which corresponded to the highest clinically employed concentrations [42,66-69]. Specifically, only at a concentration of 400 μM did phenobarbital significantly reduce the auto-correlation of SPWs (Fig. 7B), significantly increase the half-width (Fig. 7C) and significantly reduce the quantity of ripples (number, duration and power, Fig. 8). At the very high concentration of 1.6 mM abolishment of the spontaneous activity was observed (n = 3).

## Discussion

The present findings demonstrate that thiopental, at clinically relevant concentrations known to impair memory, produces a significant reduction in the incidence, rhythmicity and synchrony of hippocampal SPWs and a concomitant reduction in the quantity, but not in the frequency, of SPW-associated ripple oscillation. On the other hand, clinically relevant concentrations of phenobarbital did not significantly affect SPW-R complexes.

### Thiopental effects on in vitro SPW-R complexes

Thiopental acts in slowing or disrupting gamma [71-73], beta [61] and theta oscillations [58,65]. The present study examined, for the first time, the effects of the drug on both SPWs and ripple oscillation. Previous in vivo studies have shown that the barbiturate pentobarbital slows down or abolishes SPWs at pre-anesthetic or anesthetic doses respectively [12,74]. The general anesthetic methohexital displays analogous effects on in vitro SPWs [75], and the inhaled anesthetic halothane prevents the appearance of ripples [48]. We observed that thiopental induced concentration-dependent slowing and disorganization of SPW-Rs, resulting apparently in abolishment of the spontaneous activity at high drug concentrations. Thiopental strongly reduced the rate of SPWs and significantly prolonged single SPWs at concentrations producing sedation (~50–150 μM) [53,54] and anesthesia (>150 μM) [49,53-55], but see also [50].

SPWs generation depends on excitatory and inhibitory synaptic transmission mediated by glutamatergic non-NMDA [28,29,31,62,76] and GABA_A _receptors [28,29,62] respectively, and intense activation of inhibitory interneurons has been observed in vivo [15,47,48,77] and in vitro [78]. SPW-associated ripple oscillation is a high-frequency (100–200 Hz) field event generated from the interaction between pyramidal cells and inhibitory interneurons [8,16,46-48,79], with proposed prominent role of the interneurons [46-48].

At the cellular level, thiopental enhances GABA_A _receptor-mediated phasic inhibitory events [58-60,80-82], characteristically prolonging them [58-60,80,81]. The degree of drug-induced prolongation of inhibitory postsynaptic currents or potentials (IPSC/P) has been correlated to the degree of frequency reduction of those network oscillations, like theta [58], gamma and beta [60,61,71], whose period is at the range of IPSC duration. SPWs occurring every ~0.5 sec, display a period longer than the duration of fast IPSPs, making thus less likely the involvement of enhanced GABA_A_-mediated phasic inhibition in the drug-induced reduction of the rate of SPWs. However, as suggested by recent data [83], the important implication of phasic GABAergic transmission in the initiation of SPW-R events should be taken into account and therefore its role on the rate of SPWs should not be ruled out. At concentrations ≥50 μM thiopental enhances GABA_A _receptor-mediated tonic inhibition in hippocampal pyramidal cells [58,71] and interneurons [56], and directly activates GABA_A _receptors [58,82]. Enhancement of tonic inhibition affects neuronal excitability [56,84-86], and modeling data implicates tonic inhibition in slowing network oscillations [61,72]. Moreover, slowing of SPWs occurs under conditions of reduced neuronal excitability [87]. In the present study, thiopental reduced neuronal excitability, as observed by the reduction of unit activity. Therefore, the enhancement of tonic inhibition and the direct activation of GABA_A _receptors appears a likely explanation for the slowing down of SPWs. Thiopental could affect the rate of SPW-R activity by disturbing excitatory synaptic transmission, since slowing or even abolishment of SPWs has been observed whenever excitatory synaptic transmission is reduced [29], (Papatheodoropoulos, unpublished observations). However, we did not observe any significant drug effect on the fEPSPs.

Thiopental prolongs IPSPs in pyramidal neurons and in the present study the drug significantly prolonged SPWs. During in vitro SPWs both depolarizing and hyperpolarizing components are observed in principal neurons [31,45,62,63], although in CA3 pyramidal cells some authors have observed only depolarizing potentials [75]. In our model the most prominent intracellular correlate of SPWs in CA1 pyramidal neurons is a GABA_A _receptor-mediated hyperpolarization [28]. Thus, the prolongation of SPWs induced by thiopental might involve to some extends prolongation of GABA_A _receptor-mediated IPSPs in pyramidal cells. In this line, the absence of thiopental effect on the amplitude of SPWs is consistent with the observation that the drug, similarly to other barbiturates, does not affect the amplitude of GABA_A _receptor-mediated synaptic events [58-60,80], but see also [82].

In similarity to the previously observed disruptive effects of thiopental on rhythmicity and synchronization of in vitro theta, gamma and beta oscillations [58,61,72], at clinically relevant concentrations (≥25 μM), thiopental strongly suppressed rhythmicity and spatial synchronization of SPWs. The high degree of synchronization is actually one of the major common characteristics between in vivo [12,88] and in vitro SPWs [27-30], whereas rhythmicity characterizes mostly the in vitro [31] (Fig. 1E) rather than the in vivo SPWs [12]. Disruption of synchrony of network oscillations along the CA1 region occurs as a result of the combination of the prolongation of GABA_A _receptor-mediated synaptic events with enhanced tonic inhibition [72]. Thus, the thiopental-induced enhancement of GABA_A _receptor-mediated phasic and tonic conductances might also underlie the drug effects on synchrony and rhythmicity of SPWs.

Ripple oscillation was affected by thiopental at concentrations ≥25 μM. During ripple oscillation certain types of interneurons and a small fraction of pyramidal cells increase their activity following a temporally very precise firing pattern [46-48,77]. Therefore, excitability changes in these neurons would predict alterations in ripple oscillation characteristics. Among interneurons, bistratified and basket cells selectively fire during the whole ripple episode [77]. Thus, presumably the activity of these interneurons determines the duration of ripple episodes. We hypothesized that thiopental-induced reduction of excitability in bistratified and basket cells is critically involved in the drug effects on ripple oscillation. Interestingly, recent evidence [89] suggests that these particular types of interneurons (i.e. bistratified and basket cells) are at a relatively higher state of tonic inhibition compared to other types of inteneurons. Hippocampal interneurons but not pyramidal cells possess a baseline GABA_A _receptor-mediated tonic inhibition [85]. It might then be the case that interneurons, compared with pyramidal cells, display a greater susceptibility to thiopental-induced reduction in excitability through enhancement of tonic inhibition. This is consistent with the lower drug concentration needed to reduce ripple quantity (duration and number) compared with SPW's rate and duration. Furthermore, it supports the important role of interneurons in normal ripple generation, although oscillations at the ripple's frequency range can be observed under experimental conditions of disinhibition [83,90,91].

### Comparison of effects between thiopental and phenobarbital

The distinct effects of the two drugs on SPW-R activity, achieved at clinically relevant concentrations, resemble their differing sedative and amnesic effects. The effects of thiopental on SPW-Rs are presumably mediated via GABA_A _receptor mechanisms, since they are achieved at concentrations falling in the range of drug interaction with the GABA_A _receptor. On the other hand, the commonly employed anticonvulsant concentrations of phenobarbital (<300 μM) [42,66-69] are lower than those for effective drug interaction with the GABA_A _receptor (>300 μM) [42,68,92-95], but see also [96]. It appears therefore that the much lower effects of phenobarbital on SPW-R activity in comparison to those of thipoental's, might reflect its actual lower impact as an enhancer of GABA_A _receptor function.

### Implications

SPW-R activity has been implicated in the processes of information transfer between hippocampus and neocortex subserving memory consolidation [17,19,22,88,97]. Thiopental-induced impairment of explicit memory occurs at drug serum concentrations less than 30 μM [1,35,98]. Interestingly, the lowest concentration of the drug interfering with ripples and synchrony and rhythmicity of SPWs (25 μM) actually equates to the Cp50 value for 50% explicit memory impairment [1]. Furthermore, the only concentration of phenobarbital (200 μM) which significantly reduced the quantity of ripples was comparable to that producing explicit memory loss [42]. The present results show that thiopental affects the quantitative parameters of the ripple oscillation (i.e. the number, duration and power) but not the frequency of the oscillation. Consistently, it has been recently shown that cannabinoids, which induce memory impairment, produce reduction of the power but not of the frequency of the ripple oscillation [99]. Considering the proposed role of SPW-Rs in memory consolidation [17,23,24], as well as the in vitro induction of SPW-Rs in association with long-term synaptic plasticity [31], the interfering effects of thiopental on SPW-R activity might disrupt the hippocampal network-dependent transformation of short-term into long-term memory storage. This is in line with previous data showing that the processes involved in the formation of long-term explicit memories are sensitive to anesthetic agents [36,51,100].

SPW-R complexes can be triggered by neocortical input [8,101,102]. It could therefore be argued that thiopental exerts its amnesic actions by acting on the neocortical circuit, which only secondarily influences hippocampal function. On the other hand, the in vitro generation of SPW-Rs in hippocampal slices [27,30,31,87], in the isolated whole hippocampus [29], as well as their in vivo occurrence in the grafted hippocampus [29,43] show that they are an intrinsic hippocampal activity. Furthermore, the emergence of this activity is presumably based on strengthened synapses in the same local hippocampal circuit [29,31,103]. Consequently, the present evidence suggests that thiopental mediates its amnesic effects by directly acting on the local hippocampal circuit, and thus interfering with the hippocampo-neocortical communication, as observed also in the case of theta oscillation [29,54]. Finally, the present results are consistent with the emergent consensus on the weakening role that GABAergic transmission exerts on the memory processes [104].

## Conclusion

Thiopental, presumably by disturbing the well regulated balance between excitation and inhibition, interferes with hippocampal SPW-R activity. The action of thiopental was observed at relatively low drug concentrations reported to induce mild sedation and explicit memory impairment. The drug-induced reduction in quantity but not frequency of the ripple oscillation is suggestive of the importance of the former parameter as a basic functional feature of the SPW-associated high-frequency oscillation. We hypothesize that the thiopental-induced effects on SPW-R activity are implicated in the drug-induced impairment of hippocampus-involving memory processes.

## Methods

### Slice preparation

Hippocampal slices were prepared from male Wistar rats, 4–10 weeks old, housed in a room with a controlled temperature (22–24° C), 12/12 hrs light-dark cycle. All measures were taken to minimize animal suffering and to reduce the number of the animals used, according to the European Communities Council Directive Guidelines (86/609/EEC) for the care and use of Laboratory animals. Animals were deeply anaesthetized with diethyl-ether and decapitated. The brain was removed and placed in chilled (2–4°C) artificial cerebrospinal fluid (ACSF) and the two hippocampi were excised free. Using a McIlwain tissue chopper, transverse slices (500–550 μm thick) were prepared from the ventral hippocampus, as previously described [87]. The slices were immediately transferred on the two independent channels of an interface type recording chamber and maintained at a constant temperature of 32 ± 0.2°C. In some experiments the temperature was changed in order to observe its effects on the spontaneous activity (see Results). The slices were continuously humidified with 95% O_2 _and 5% CO_2 _mixed gas and perfused at a rate of 0.8–1.0 ml/min with standard ACSF containing (in mM): 124 NaCl; 4 KCl; 2 MgSO_4_; 2 CaCl_2_; 1.25 NaH_2_PO_4_; 26 NaHCO_3_; 10 glucose; at pH 7.4, equilibrated with 95% O_2 _and 5% CO_2 _gas mixture. Drugs included thiopental (Pentothal, Abbott Laboratoires, S.p.A. Italy) phenobarbital (Sigma, USA) and the NMDA receptor antagonist 3-((*R*)-2-Carboxypiperazin-4-yl)-propyl-1-phosphonic acid (CPP, Tocris, UK). All drugs were prepared as stock solutions and then were dissolved in ACSF and bath applied. CPP was applied for at least fifteen minutes before the application of thiopental. Thiopental and phenobarbital were applied for thirty minutes. In some experiments thiopental was applied for longer periods, as indicated in figures.

### Electrophysiological Recordings

Field potentials were recorded from CA1 or CA3 stratum pyramidale or st. radiatum using carbon fibre electrodes, of a diameter either 7 μm (homemade) or 10 μm (World Precision Instruments Inc., USA). A bipolar platinum/iridium electrode (25 μm) located in stratum radiatum was used for electrical stimulation of the Schaffer collaterals (pulses: 0.3–0.9 mA/0.1 ms). Spontaneous field potentials were gradually organized in the ventral hippocampal slices during the first 2–3 h of their maintenance in the recording chamber, under standard conditions. Spontaneous activity was fully organized in terms of amplitude and frequency after about three hours from slice placement on the chamber (Fig. 1A). Stabilized activity was recorded constantly for at least three hours thereafter without interruption or considerable decline in amplitude and frequency of occurrence. Activity in many slices was stable for more than seven hours. The consistency over time (i.e. the stability of phenomenological characteristics, like frequency and amplitude) allows the detailed examination of pharmacological effects over a wide time scale. In order to determine the degree of synchrony of sharp waves (SPWs) across CA1 st. pyramidale two simultaneous field recordings were made along the pyramidal layer at a fixed distance of 1 mm.

### Data processing, Measurements and Analysis

Signals were band-pass filtered at 0.5 Hz – 2 kHz using a Neurolog amplifier system (Digitimer Limited, UK), and a Grass AC/DC amplifier when double simultaneous recordings were performed. Data were digitized at 4 kHz and stored in a computer disk using the CED 1401-plus interface and analyzed off-line using the Spike2 and Signal software (Cambridge Electronic Design, Cambridge, UK).

Spontaneous activity in stratum pyramidale consisted of a positive slow wave consistently ridden by a burst-like high-frequency activity. The latter was mainly manifested during slow wave's rising and peaking phase (Fig. 1C1). Measures of the spontaneous activity were performed on the slow and high frequency components, which were disclosed after filtering original recordings at different frequency windows (Fig. 1C-2,C3 and 1C4). Specifically, SPWs were detected after low-pass filtering the original signal at 35 Hz and setting a threshold at a level in which all putative events, verified by visual inspection, were marked. In some experiments, in which the amplitude of SPWs was greatly reduced during the pharmacological procedure, adjustment of the threshold was required in order to make the detection of all events in the record reliable. SPWs were quantified by the following measures: a) period, determined as the time interval between the peaks of successive waves; b) amplitude, determined as the voltage difference between the positive peak and the baseline; c) duration, determined as the time elapsed between the two points of intersection of the field positive waveform with the baseline; d) half-maximal width, determined as the time elapsed between the two points of intersection of the field waveform with the horizontal line passing through the half-maximal amplitude of the wave. Measures of duration and half-maximal width were performed on the average sweep calculated from at least thirty single events; e) auto-correlation, used as an index of the rhythmicity of SPWs, was estimated by the amplitude of the first side-peak of the auto-correlogram [105]; f) cross-correlation, measured by the peak amplitude of the corresponding cross-correlogram calculated using simultaneous recordings from two slice locations with a distance between them of 1 mm. Cross-correlation was used as an index of the synchronization of SPWs along the pyramidal layer. The measures of period, amplitude and duration of SPW were made from two minute long epochs of low-pass filtered record. Auto-correlation and cross-correlation were obtained from a recording epoch lasting ten to twenty seconds. Ripples were detected after band-pass filtering original records at 80–300 Hz (1C-3) and setting a threshold at four times the standard deviation of event-free baseline noise. Furthermore, events were categorized as ripples only when episodes of at least three consecutive negative deflections were observed with delays between them of at least 2 ms and no more than 11 ms, i.e. they should have a frequency falling within the range of 90–500 Hz [62]. Threshold was further verified by visual inspection. Ripples were therefore quantified by their: a) number of ripples inside a ripple event, estimated by the number of negative deflections inside an event; b) duration of the ripple event; c) intra-ripple frequency, determined as the reciprocal of the value of the mean inter-ripple interval; d) amplitude, determined as the voltage difference between the positive and negative maximum in each ripple event. Measures of ripple characteristics from each slice or condition were made from thirty consecutive ripple events. Ripple power and frequency were additionally verified from power spectra applied to five minute long raw record. Multiunit activity was revealed by band-pass filtering data at 500–1000 Hz, and the negative spikes were detected setting the threshold level four times the standard deviation of event-free baseline noise.

Examination of drug effects on SPW-R activity was performed only on those slices displaying stable activity for at least fifteen minutes. For that, the power of ripples and the auto-correlation in SPWs were on-line analyzed and monitored in a continuous manner. Whenever recordings were made from two locations in the same slice, cross-correlation analysis was also continuously monitored.

Values through the text are expressed as mean ± S.E.M., and "n" indicates the number of slices included in the analysis. For statistical comparisons of the effects of thiopental and phenobarbital on SPW-Rs, measures were taken before drug application and thirty minutes after drug application. We used SPSS 14 for statistical analysis (paired and independent t-tests), Origin 7 to make figures and Reference Manager 10 to construct the list of references.

## Authors' contributions

CP conceived of the study, made the experimental design, carried out most of the experiments, participated in the data analysis and wrote and prepared the manuscript. ES participated in some experiments and contributed to the data analysis. DK contributed to the data analysis. PD participated in the experimental work, contributed to the data analysis of most of the experiments and helped with the data archiving. All authors read and approved the final manuscript.
